# A Unified Theory for the Development of Tinnitus Perception and Hyperacusis Based on Associative Plasticity in the Dorsal Cochlear Nucleus

**DOI:** 10.3390/brainsci16040395

**Published:** 2026-04-04

**Authors:** Holger Schulze, Achim Schilling

**Affiliations:** 1 ENT Clinic Head and Neck Surgery, Experimental Otolaryngology, University Hospital Erlangen, Waldstrasse 1, 91054 Erlangen, Germany; 2 Center for Neuromodulation and Neuroprosthetics, University Hospital Mannheim, University Heidelberg, Theodor-Kutzer-Ufer 1-3, 68167 Mannheim, Germany; achim.schilling@medma.uni-heidelberg.de; 3 BG Clinic Ludwigshafen, Ludwig-Guttmann-Straße 13, 67071 Ludwigshafen, Germany

**Keywords:** Hebbian plasticity, classical conditioning, hearing disorder, phantom perception, stochastic resonance

## Abstract

**Background/Objectives**: Tinnitus and hyperacusis can occur together or in isolation, with hyperacusis being associated with tinnitus much more frequently than vice versa. This striking correlation between tinnitus and hyperacusis prevalence implies that there might be a common origin, such as (hidden) hearing loss, and possibly interrelated neural mechanisms in the pathological development of those two conditions. Here, we propose such interrelated pathological mechanisms. **Methods**: This is a theoretical work based solely on considerations and published data. **Results**: We propose a model localized in the dorsal cochlear nucleus (DCN) of the brainstem, based on classical mechanisms of Hebbian and associative plasticity known from classical conditioning. Specifically, our model proposes that hyperacusis results from the synaptic enhancement of cochlear input to the DCN, whereas chronic tinnitus results from the synaptic enhancement of somatosensory input to the DCN. Specific conditions leading to one or the other condition are discussed. **Conclusions**: Our model predicts that hearing loss leads to chronic tinnitus, while noise exposure (which may also cause hearing loss) leads to hyperacusis. We would like to emphasize that our aim with the proposed model is not to provide a self-contained theoretical construct, but to stimulate thought regarding possible pathological causes of tinnitus and hyperacusis that have not yet been investigated. Individual assumptions that cannot yet be substantiated by the existing literature are intended to provide impetus for future experimental studies.

## 1. Introduction

Subjective tinnitus is defined as the perception of a sound in the absence of any physical sound source [1] and often leads to an enormous psychic burden [2]. In Europe, the prevalence of tinnitus ranges from 8.7% to 28.3%, with a prevalence of 12% in Germany [3]. Approximately 2% of tinnitus patients severely suffer from tinnitus and related comorbidities such as concentration issues, stress, and depression [2,4]. Interestingly, 30% to 80% of tinnitus patients suffer from another pathological condition, namely hyperacusis [5,6]. Hyperacusis refers to a reduced tolerance to sounds that are perceived as normally loud by the majority of the population [7]. However, at present there is no commonly accepted scientific definition [7]. In contrast to tinnitus patients, where 8% to 28% suffer from hyperacusis, only 5% to 10% of the general population suffers from hyperacusis [6]. Furthermore, 86% of hyperacusis patients suffer from tinnitus [8]. In other words, patients may suffer exclusively from tinnitus, exclusively from hyperacusis, or from both pathological conditions. The fact that both conditions can occur in isolation suggests that tinnitus and hyperacusis are caused by disjoint neural mechanisms [9]. However, the striking correlation between tinnitus and hyperacusis prevalence implies that there might be a common origin, such as (hidden) hearing loss [10,11,12], and possibly interrelated neural mechanisms of pathological development [9].

In this theoretical paper, we propose interrelated pathological mechanisms, localized in the brainstem, based on classical mechanisms of Hebbian and associative plasticity known from classical conditioning. Note that our model merely explains how the pathophysiological activity that underlies tinnitus and hyperacusis first develops in the peripheral auditory system. No predictions or assumptions are made regarding possible mechanisms upstream the dorsal cochlear nucleus (DCN), such as gating problems in the thalamus [13], the conscious cortical perception of a phantom sound [14,15,16], or burden. Therefore, our model also does not address the distinction between tinnitus perception and tinnitus disorder, as these are based on mechanisms further upstream the auditory pathway and associated neural networks, such as limbic structures or the salience network, but rather focuses on peripheral pathophysiological neural activity development. Furthermore, we will use the term hyperacusis in the sense of recruitment [8,17], which refers to the characteristic feature that mild sounds are over-amplified along the auditory pathway and perceived as too loud [9,18]. The model presented here is a further development of the Erlangen Model of Tinnitus Development.

### 1.1. The Erlangen Model of Tinnitus Development

**Figure 1:**
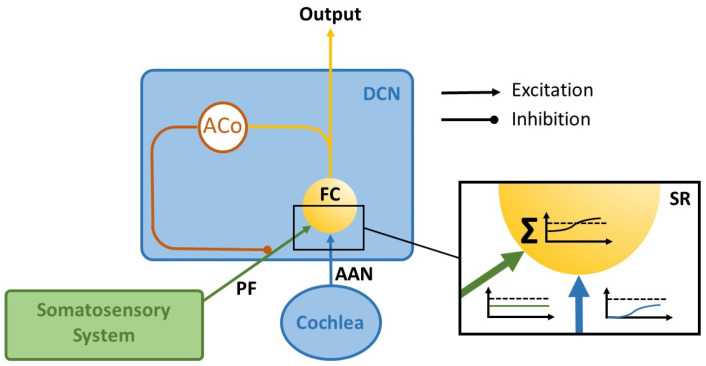
The Erlangen Model of Tinnitus development: AAN = auditory afferent nerve; ACo = Autocorrelation; FC = fusiform cell; PF = parallel fiber; SR = stochastic resonance. Figure adopted from [29]. For explanation, refer to the text.

There is a broad consensus that tinnitus, as well as hyperacusis, are induced by some kind of hearing loss [11,12,19]. This hearing loss is based on a reduction in the innervation of the inner hair cells of the cochlea [20], which can be so slight that no increase in the hearing thresholds can be measured in the audiogram (“hidden hearing loss”, cf. [21]). However, the result is still a reduced input from the cochlea into the DCN, which can lead to weak signals no longer triggering a supra-threshold reaction from the corresponding neurons in the DCN and thus no longer being transmitted to the subsequent auditory pathway. Additionally, tinnitus is characterized by an increase in spontaneous neural activity along the auditory pathway, starting at the DCN [18,22]. However, tinnitus is not related to an increased stimulus-evoked activity, whereas hyperacusis in contrast is indeed characterized by increased stimulus-evoked activity [23,24]. Additionally, it is well known that tinnitus can occur within seconds of short transient hearing loss [25], which is far too fast for plastic adaptations like homeostatic plasticity or so- called central gain mechanisms [10], a comparatively slow process over timescales of hours or days [26,27]. In 2016, we proposed a mechanism based on a simple feedback loop that can cause tinnitus-related hyperactivity in the brainstem (The Erlangen Model of Tinnitus Development, [28,29]). The idea of that mechanism (cf. Figure 1) is that a signal coming from the cochlea (blue) which is subthreshold, e.g., due to hearing loss, is lifted above the detection threshold (dashes line) by means of stochastic resonance (SR), that is, by adding neural noise which is believed to come from the somatosensory system (green, cf. [30]) to the cochlear input. In this way, the transmission of weak signals into the auditory pathway is enabled again, and thus hearing is improved overall. This improvement in hearing is believed to be fine-tuned at the millisecond scale and to constantly operate, even in the healthy auditory system. In cases of tinnitus, the neuronal somatosensory noise that is added to the cochlear input itself is strong enough to cross the threshold of the fusiform cells (FC) of the DCN and is therefore perceived as tinnitus. In other words, from the perspective of the Erlangen model, tinnitus is a by-product of a mechanism designed to constantly optimize the hearing process.

**Figure 2:**
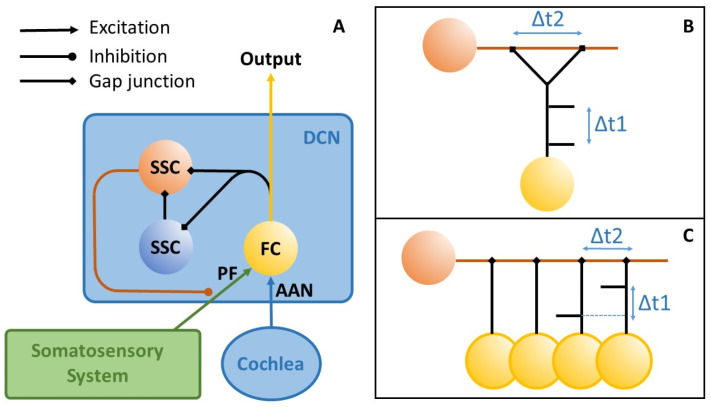
Possible implementations (**A**–**C**) of delay lines for calculating the autocorrelation function within the DCN. (**A**): Anatomically plausible DCN neuronal network to detect a given delay. References for the proposed circuitry: [33,34,35,36,37]. Note that the autocorrelation function may, in principle, also be calculated on the dendrites of an SSC (**B**,**C**): If Δt1 (time difference between two spikes) equals Δt2, a spatial summation of excitatory postsynaptic potentials (EPSPs) would occur that may drive the SSC. This can be achieved by either copying the input of a single FC to two different locations on the same dendrite (**B**) or by converging the input of several FCs within a given frequency channel to the same SSC dendrite (**C**). Note that, in the latter case even, Wever‘s Volley Theory could allow for Δt intervals below 1 ms [38].

To achieve this optimization, the amplitude of the neural somatosensory noise that is added to the auditory input is fine-tuned through a feedback loop that maximizes information transmission into the auditory system by maximizing the autocorrelation function (ACo) and, by this means, the information content of the DCN output [28,29,31]: If this amplitude is too low, the sum of the cochlear and somatosensory input does not reach the threshold of the DCN neurons; if it is too high, the signal is masked by the noise. According to our model, the DCN uses so-called delay lines [32] for this purpose, which superimpose a signal on themselves with a time delay and can thus recognize regularly occurring patterns at fixed time intervals (which are not to be expected in noise). Unlike Licklider’s original model, the delay line in our context does not need to detect any delay (which would require a series of coincidence detectors representing different time delays), but only one coincidence detector per frequency channel, since the expected time delay corresponds to the characteristic frequency of that channel (cf. Figure 2). Therefore, the output of the yellow neuron is fed directly to a coincidence detector (red neuron) with a frequency-specific time delay corresponding to the time interval that is to be detected (via the blue delay neuron). The red neuron only reacts if it simultaneously receives input from the direct and the time-delayed projection, i.e., at the exact point when two action potentials occur with the desired time interval, which corresponds to the characteristic frequency in the respective frequency channel.

To provide a rough estimate as to whether the computation of an autocorrelation function could plausibly be supported by dendritic processing in the human dorsal cochlear nucleus, we consider the following. The calculation of the autocorrelation value needed for the SR effect requires, for a signal frequency f, a time delay of 1/f, e.g., for a 1 kHz tone, a time delay of 1 ms is needed. Assuming an average dendritic conduction velocity of approximately 0.5 m/s, as measured by Larkum and co-workers in 1996 [39], and that the time delay is exclusively generated through differences in dendritic path length, a length difference of approximately 8 mm would permit frequencies to be processed down to 62 Hz. The approximate size of the longest dendrites in the cat DCN is 8 mm [40]. For a 20 Hz tone, a length difference of 25 mm would be needed. Thus, for lower frequencies, an intermediate neuron/synapse might be necessary. By contrast, calculating the autocorrelation of a 5 kHz signal would require length differences of only 100 µm, demonstrating that the upper frequency range is not limited by dendritic lengths but rather by the precision of the spike timing of the input signal. AAN = auditory afferent nerve; FC = fusiform cell; PF = parallel fiber; SSC = superficial stellate cell. The Figure was adopted from [29].

For a characteristic frequency of 1 kHz, for example, this interval would be one millisecond. Gap junctions, together with the adjusted fiber length within this feedback loop, allow for fine-tuning to the desired interval. Finally, the coincidence detector, in turn, inhibits the somatosensory input. The greater the information content of the DCN output, the less noise from the somatosensory system has to be added to the cochlear input to ensure optimal information transmission. Note that our model does not rule out the possibility that there may be other mechanisms underlying the development of tinnitus that are independent of the DCN.

### 1.2. Classical Conditioning

In classical conditioning, the association between an unconditioned stimulus (US) that is able to induce a naturally occurring unconditioned response (UR) and a conditioned stimulus (CS) is learned. In Pavlov’s original work, the salivation of a dog is the UR that follows the presentation of food (US). The ringing of a bell (CS) is able to induce the same response if the CS is repeatedly presented prior to the US [41]. This response to the bell is therefore called conditioned response (CR). Here, we now incorporate the mechanistic principles known from classical conditioning into our model, so that it is not only able to explain the initial development of acute tinnitus perception, but also the pathological conditions of chronic tinnitus and hyperacusis at the level of the DCN. Here, we make no statements on higher brain structures and top-down mechanisms.

## 2. Materials and Methods

This is a theoretical work based solely on considerations and published data.

## 3. Results

**Figure 3:**
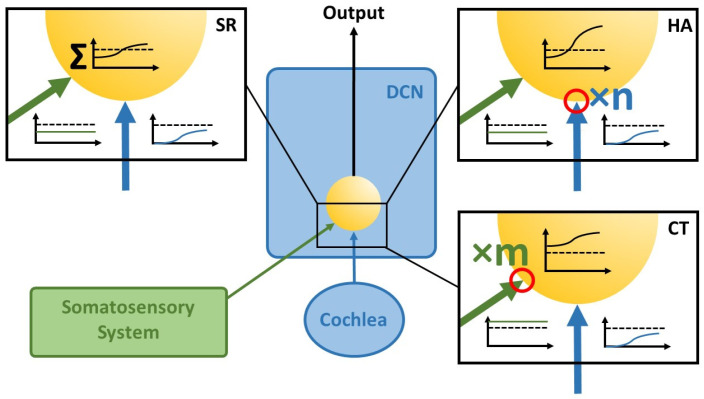
Graphical abstract of the proposed theory. The theory is based on activity-dependent plastic changes in the synaptic weights of inputs from the somatosensory system (i.e., the trigeminal ganglion and spinal trigeminal nucleus, [30]) and the cochlear nerve to the DCN. The increased synaptic strength (red circles) of the cochlear input is responsible for HA, while the increased synaptic strength of the somatosensory input is responsible for CT. DCN = dorsal cochlear nucleus; SR = stochastic resonance; HA = hyperacusis; CT = chronic tinnitus. For further explanations, refer to the text.

In this theoretical paper, we put forward the hypothesis that the Erlangen model for tinnitus development, described in Figure 1 and Figure 2, is also able to explain the development of hyperacusis. Our hypothesis is based on the notion that the essential connectivity pattern of two converging inputs into the DCN, namely, one from the cochlea and one from the somatosensory (trigeminal) system (cf. Figure 1), mimics the basic neuronal circuit known from classical conditioning. The central idea of the theory is that—analogously to classical conditioning—the weights of these inputs to the DCN can be enhanced by means of synaptic plasticity triggered by certain input conditions, and especially the relative timing between them. As a result, amplification of the cochlear input to the DCN would result in hyperacusis (HA, Figure 3, upper-right panel) while amplification of the somatosensory input would lead to chronic tinnitus (CT, Figure 3, lower-right panel). While such plasticity has already been demonstrated for the somatosensory input, evidence for such plasticity in the auditory input is still pending [42].

### 3.1. Development of Chronic Tinnitus

**Figure 4:**
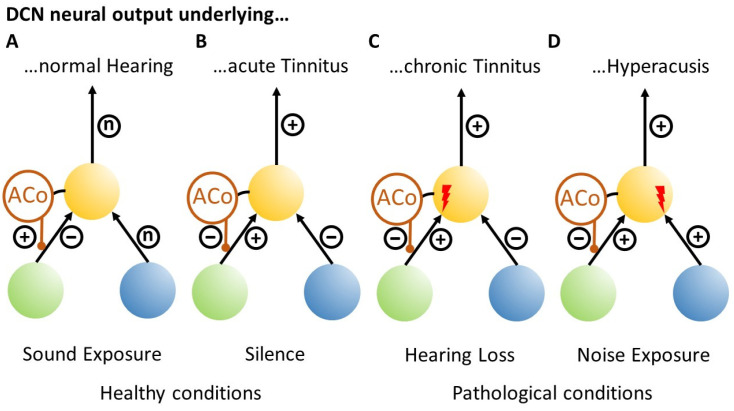
The Erlangen Unified Model of Tinnitus and Hyperacusis Development. The panels show simplified sketches of the connectivity scheme presented in Figure 1. Yellow ball: DCN input neuron; blue ball: cochlear input to the DCN; green ball: somatosensory input to the DCN; ACo: autocorrelation detector; black lines: excitatory connections; red lines: inhibitory connections; red flash: synaptic strengthening; n = normal activity; + = increased activity; − = reduced activity. For further explanation, refer to the text.

In our Erlangen model of tinnitus development, we described how tinnitus-related hyperactivity develops by means of stochastic resonance (cf. Figure 1), but we have not yet explained how the initial occurrence of such hyperactivity, i.e., acute tinnitus, can eventually lead to the condition of chronic tinnitus. In our model, acute tinnitus (Figure 4B) occurs if the cochlear input to the DCN is reduced. This could be the case, for example, in complete silence in an anechoic chamber, where people experience transient tinnitus as long as they remain in silence. In the clinically relevant case of reduced cochlear input due to hearing loss, chronic tinnitus (Figure 4C) may develop if the hearing loss is permanent. In this case, according to our model, the reduced cochlear input leads to a reduction in information transmission into the auditory system, which is detected by the DCN circuit in the form of reduced autocorrelation of the DCN output (cf. Figure 1 and Figure 4C). The reduced autocorrelation causes a disinhibition of the somatosensory input to the DCN.

In classical conditioning, the conditioned stimulus (CS) must precede the unconditioned stimulus (US) in order to induce the synaptic plasticity that results in conditioned responses (CR), i.e., strengthens the synaptic weight of the CS input [41]. When applied to our model, this means that if cochlear input (analogous to the US) that is reduced due to permanent hearing loss reaches the DCN neuron that is already activated by the disinhibited somatosensory input (analogous to the CS), then the synaptic weight of the somatosensory input would be increased (Figure 3, xm; Figure 4C, red flash). As a result, this input alone becomes strong enough to drive the DCN neuron above the threshold, generating an output that is transmitted upstream into the auditory pathway and can finally be perceived as tinnitus (analogous to the CR).

### 3.2. Development of Hyperacusis

If we now take this model further, hyperacusis (Figure 4D) can develop if the cochlear input to the DCN is amplified rather than the somatosensory input (Figure 3, xn; Figure 4D, red flash). For this to happen, the relative timing of the two inputs is crucial [41]. This means that if the cochlear input is to be amplified, it must precede the somatosensory input. This would occur in case of continuous noise exposure, where the cochlear input is constantly activated, but due to the uncorrelated nature of the noise, the autocorrelation of the DCN output would still be low, which also leads to disinhibition of the somatosensory input. Therefore, in contrast to the permanent hearing loss condition described above, where the activation of somatosensory input precedes cochlear input, this relative timing of inputs to the DCN would be (statistically) reversed in the case of continuous noise exposure, resulting in an increase in the synaptic weight of the cochlear input and consequently hyperacusis.

## 4. Discussion

Tinnitus and hyperacusis can occur separately or together, which is easily explained by our model as it links the two pathologies to the plastic amplification of two separate synapses: according to the model, amplification of the cochlear input to the DCN leads to hyperacusis, while amplification of the somatosensory input to the DCN leads to tinnitus. In tinnitus, these changes during an acute phase depend on the autocorrelation of the output activity of the fusiform cells, which reflects information content and which is calculated within the DCN to modulate the input from the somatosensory system via a feedback loop (cf. Figure 1). Once this somatosensory input is permanently amplified by long-term potentiation (LPT), as described in our extension of the Erlangen model presented here, acute tinnitus would turn into chronic tinnitus.

In cases where both inputs to the DCN, the somatosensory and the auditory, are amplified, tinnitus and hyperacusis occur together. In such cases, interestingly, when both conditions co-occur, increases in sound-evoked activity can be found throughout the auditory pathway [23,43]. This phenomenon is also consistent with our model, as the offset shift and the greater steepness of the rate intensity functions (Figure 3, insets on the right) in tinnitus and hyperacusis are superimposed. In patients who only have tinnitus but no hyperacusis, this superimposition, and thus the additional amplification factor of the cochlear input, is missing. As our model predicts, the two pathologies may reinforce each other, which is consistent with clinical data [44,45].

Interestingly, a recent paper on tinnitus and hyperacusis in rats [46] reported that tinnitus symptoms were typically correlated with responses to low-intensity tones, while neural correlates of hyperacusis were found in response to moderate- and high-intensity tones. This observation—although made at the level of the auditory cortex—would be consistent with our model, since it presents tinnitus as based on stochastic resonance in the DCN, which raises low-level signals above the neural response threshold of fusiform cells, while hyperacusis presumably further amplifies signals that would be above the threshold anyway.

Central to the unified theory of tinnitus and hyperacusis development presented here is the hypothesis that both the development of tinnitus and hyperacusis are based on synaptic reinforcements—i.e., LTP. In favor of this view is the finding that in bimodal neurons in the DCN, that is, those (fusiform) neurons that receive both auditory as well as somatosensory input, an increase in response amplitudes to somatosensory input was observed after noise-induced hearing loss in guinea pigs [47]. Furthermore, the same study reported an enhancement of bimodal integration in those cells.

A direct and testable prediction from our model is that no tinnitus should develop if the development of LTP in the DCN was prevented. Specifically, tinnitus should not develop if the LTP at the somatosensory input synapses were blocked. In fact, it has already been shown that this does indeed appear to be the case: Tagoe and colleagues [48] have demonstrated that blocking LTP at a parallel fiber input to DCN using an NMDA receptor antagonist or by increasing extracellular calcium concentration reduces behavioral signs of tinnitus in rats after acoustic over-exposure. The fact that NMDA receptor blockers have not yet proven effective in clinical tinnitus therapy [49,50,51] may be due to the fact that, according to our model, their use should only be effective in an early acute phase of tinnitus development and that local administration to the DCN might be necessary.

Finally, we would like to note again out that our model makes no assumptions about what happens along the auditory pathway beyond the DCN, since there are other models published that deal with these higher-order phenomena, such as the predictive coding model [14,52] or gating models that involve the limbic system [13,53]. Our model therefore only explains how the pathophysiological neuronal activity initially develops in the periphery, which may then be perceived as tinnitus (or hyperacusis) when it is propagated to the auditory cortex. If it is not propagated to the auditory cortex, e.g., due to some kind of gating mechanism, tinnitus is not perceived even in the presence of the pathophysiological activity in the peripheral auditory pathway. This view also explains why not all patients with hearing loss experience tinnitus. An overview of how the various peripheral and central mechanisms involved in tinnitus may be related to each other can be found in the work of Schilling and colleagues [9].

## 5. Conclusions

Our model offers a new, very simple explanation for the development of the neuropathologic activity underlying both chronic tinnitus and hyperacusis and is able to describe possible interactions between the two pathologies. To the best of our knowledge, our theory is the first that links and mechanistically explains LTP in the DCN with principles of classical conditioning and the development of tinnitus and hyperacusis. As far as we know, it is also the first model that describes a developmental mechanism purely in the DCN and without the involvement of higher brain areas. The fact that the plastic synaptic changes postulated here are based on mechanisms that are analogous to known learning phenomena such as classical conditioning potentially opens up the possibility of specifically reversing the pathophysiological processes described. Since tinnitus and hyperacusis, as detailed above, are not solely attributable to the initial development of pathophysiological activity in the peripheral auditory pathway, as proposed here, but also involve neuroplastic processes in central brain regions during chronic manifestation, it is unclear whether reversing the peripheral plastic processes could also reverse tinnitus and/or hyperacusis or decrease their perception or burden. Nevertheless, triggering such reverse plasticity could be an approach that might contribute to a genuine cure for tinnitus and hyperacusis. One conceivable approach could be to revive well-known concepts such as Jastreboff’s tinnitus retraining strategy [54] in an adapted form, to develop pharmacological interventions aimed at blocking LTP in the DCN in a critical time-window after noise trauma, or to LTD at the respective DCN input synapses after tinnitus and/or hyperacusis has become chronic.

## Data Availability

No new data were created or analyzed in this study. Data sharing is not applicable to this article.

## Abbreviations

The following abbreviations are used in this manuscript:

AAN	auditory afferent nerve
ACo	autocorrelation
CR	conditioned response
CS	conditioned stimulus
CT	chronic tinnitus
DCN	dorsal cochlear nucleus
EPSP	excitatory postsynaptic potential
FC	fusiform cells
HA	hyperacusis
LTD	long-term depression
LTP	long-term potentiation
NMDA	N-methyl-D-aspartate
PF	parallel fiber
SR	stochastic resonance
SSC	superficial stellate cell
UR	unconditioned response
US	unconditioned stimulus
